# Common and uncommon lesions of the vulva and vagina on magnetic resonance imaging: correlations with pathological findings

**DOI:** 10.1259/bjro.20230002

**Published:** 2023-06-28

**Authors:** Yan Ning, Rennan Ling, Feiran Zhang, Guofu Zhang, He Zhang

**Affiliations:** 1 Department of Pathology, Obstetrics and Gynecology Hospital, Fudan University, Shanghai, China; 2 Department of Radiology, Shenzhen People's Hospital, 2nd Clinical Medical College of Jinan University, 1st Affiliated Hospital of Southern University of Science and Technology, P.R. China, ShenZhen, China; 3 Department of Radiology, Obstetrics and Gynecology Hospital, Fudan University, Shanghai, China

## Abstract

Vulvar and vaginal lesions representing a wide spectrum of diseases in female lower genital tract diseases make up a small part of all gynecological etiologies. Many of them are rare etiologies and are reported in case-reports studies. Translabial and transperineal ultrasound are modalities of choice for the first evaluation of perineal lesions. MRI is usually performed to determine the etiology of the lesions and stage. Benign lesions of the vulva and vagina usually manifest as simple cystic (vestibular cyst or endometrioma) or solid lesions (leiomyoma or angiofibroblastoma), while malignancies usually appear as large, solid masses and fill into both vaginal and perineal area. Post-contrast images play an important role in establishing a differential diagnosis, however, some benign lesions can also exhibit a vivid enhancement. Knowledge about radiologic-associated pathological manifestations may aid clinicians in better understanding these pathologies, especially for some rare lesions, and making a proper diagnosis before invasive procedures.

## Introduction

Female lower genital tract diseases, mainly including vulvar and vaginal abnormalities, represent a wide spectrum of diseases (*e.g.* benign tumors, malignancies, and developmental conditions). Vulvar cancer is the fourth most common gynecologic malignancy.^1–4^ Both translabial and transperineal ultrasound are modalities of choice for the first evaluation of perineal lesions. Compared with sonography, MRI has better soft tissue resolution and is more commonly used to outline, diagnose, and stage these conditions in clinical units. Unlike lesions in the cervix and uterus, some tiny lesions in the perineum may be overlooked even on multiplanar MRI.^5^ Thus, scrutinized review of pre-operative MRI plays a vital role in subsequent treatment design. The final diagnosis depends mainly on histological findings. This article reviews the MRI manifestations of various abnormalities of the vulva and vagina. Furthermore, the correlation of MRI with pathological findings is also comprehensively reviewed, providing its advantage in the differential diagnosis between MRI and pathological findings.

### Advanced MR scan technique and related anatomy in the pelvis

**Figure 1.**
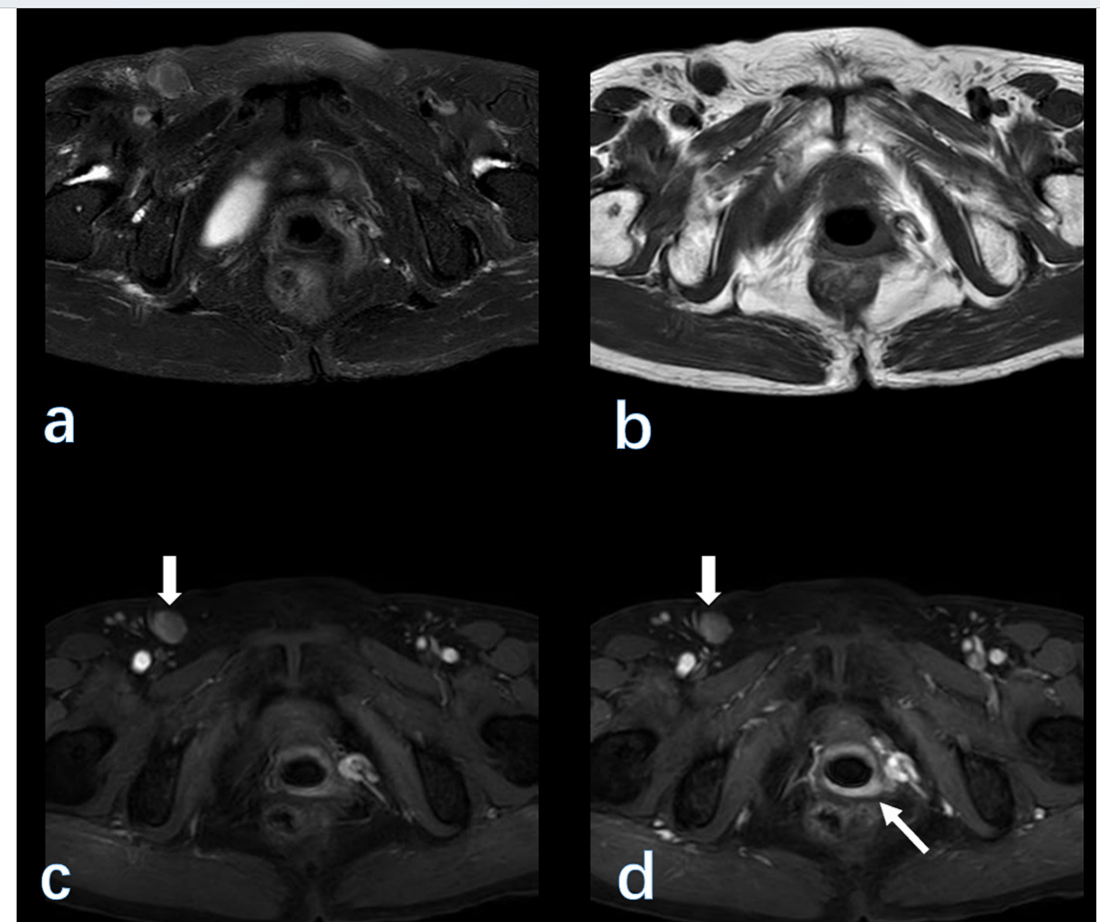
(A 72-year-old female with vaginal cancer. From (a–d) are fat-saturated *T*_2_WI, *T*_1_WI, early-contrasted, and delay-contrasted *T*_1_WI images on 3 T MRI. The vaginal wall (thin arrow) is enhanced on post-contrast images. Note, the enlarged nodes (bold arrow) in the right inguinal region.

MR is used as an imaging modality for indeterminate pelvic masses detected on ultrasound. 1.5 T MR unit is the most commonly used equipment worldwide clinically. The major limitation is sometimes low signal-noise-ratio compared with higher magnetitic field MR units. 3.0 T MR unit is also used for several decades in clinics with a more clear description of pelvis anatomy. On higher magnetitic field MR scanners (3.0 T or 7.0 T), the longer acquisition time will increase the movement-related artifacts and decrease the image quality. In our institution(Department of Radiology, Obstetrics and Gynecology Hospital, Fudan University), both equipments are applied for clinical purposes. Scanning sequences will cover conventional protocols (*T*_1_WI/*T*_2_WI with or without fat-saturation (FS) technique/DWI in either axial, sagittal, or coronal scan plane) and some complicated sequences (*e.g.* high resolution or dynamic contrast-enhanced scan) based on various suspected etiologies (Supplementary Table 1-4). Contrast material is routinely used for both vulvar and vaginal lesions during MR scans, especially for malignant lesions (Figure 1). Coronal MRI without FS technique will best depict the vaginal and vulvar anatomy. For vaginal lesions, coupling gel intravaginal or gauze filling, sometimes, is needed to better display the relationship between the lesion and surrounding tissues.

### Benign etiologies

#### Vulvar endometriosis

##### Clinical background

**Figure 2.**
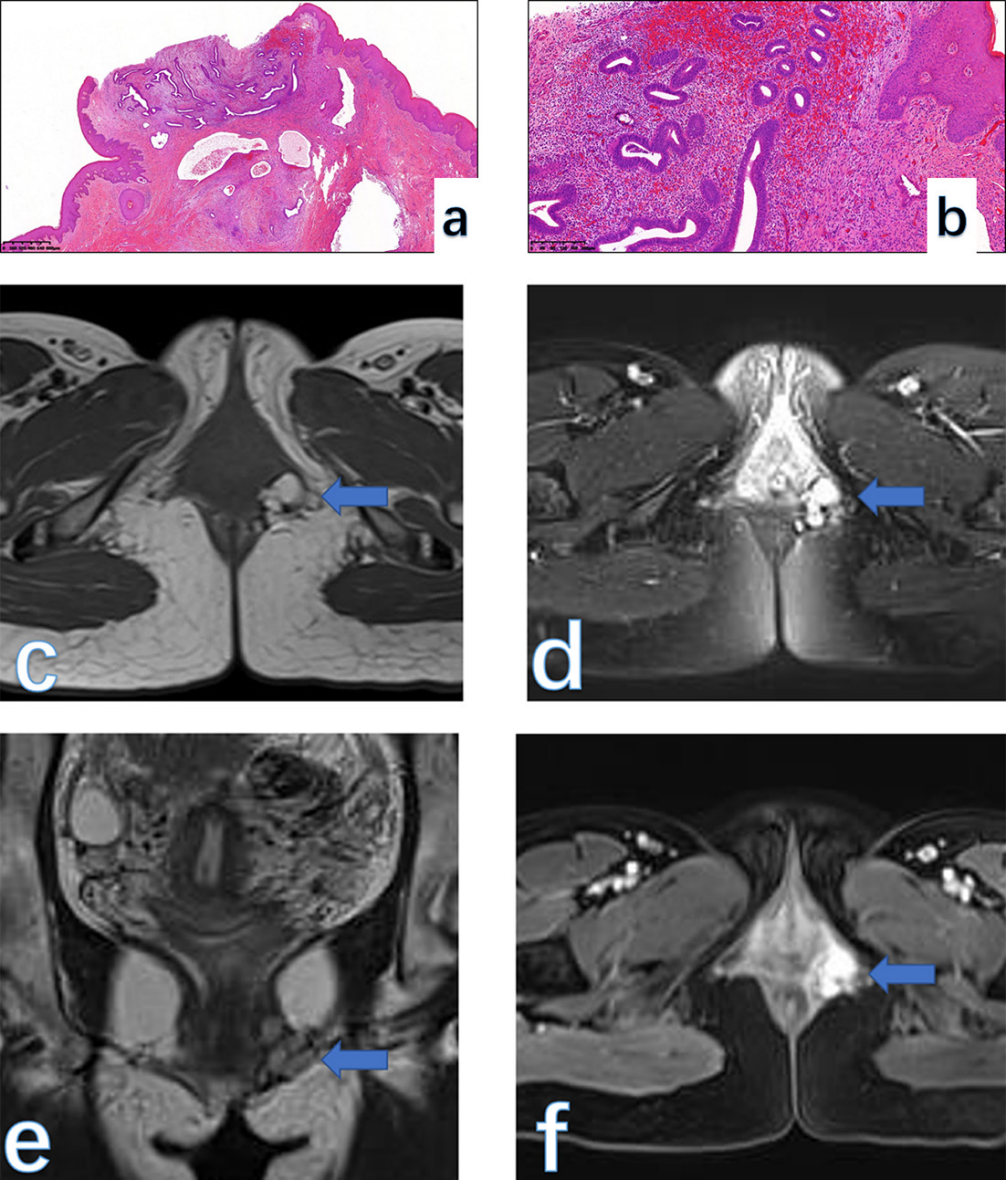
Endometrial glandular and stromal elements within the fibromuscular layer (a, HE × 25). Higher magnification of the field reveals endometrial glands surrounded by a hemorrhagic stroma within fibromuscular tissue (b, HE × 100). On MRI, in the left labium major, the multicystic lesion (arrow) with irregular margin displayed a high signal on both *T*_1_WI (**c**) and fat-suppressed *T*_2_WI (**d**) and conventional *T*_2_WI (**e**). The lesion did not enhance on the post-contrast images (**f**). The final pathological species confirmed the diagnosis of endometriosis.

Endometriosis (ES) affects approximately 10% of females of reproductive age. Endometriosis can occur in the uterus (also known as adenomyosis) or some other anatomic structures.^6^ It is usually seen in the pelvis, including the peritoneum and adnexa. However, it is uncommonly found in the bowel, ureter, and cesarean section scar. It can be detected in the cervix, vagina, and vulva.^7^ Relatively, the vagina is the most commonly affected site, accounting for 0.3–1.4% of all pelvic ES.^8^ Typical microscopic findings including both endometriotic epithelium and stroma are usually present, but a diagnosis of ES is often possible when only one of these components is found (Figure 2A–B). Polypoid ES is a rare form of endometriosis characterized by polypoid.^9^ These multiple, mucosal, or serosal masses may mimic a neoplasm on clinical, intraoperative, and gross examination. The majority of endometriosis-associated neoplasms are malignant, with endometrioid and clear cell carcinomas being the most common etiologies.^7^ Benign and borderline seromucous tumors seem to occur less frequently.

##### MRI findings

On MRI, ES often appears as an oval and cystic lesion with a clear margin. It usually appears as a high signal on *T*_1_WI, representing bleeding components. The FS-*T*_1_WI sequence is especially important because ES always shows a high signal, which is helpful to differentiate ES from other lesions with a pure high signal on *T*_1_WI. The T2-shading effect, sometimes, may not be obvious owing to small lesions displaying a homogeneously high signal (Figure 2C–F). Differential diagnosis should include a simple cyst originating from vestibular glands. A greater vestibular gland cyst (also known as Bartholin gland cyst) often shows a fluid signal on MRI, manifesting as a low signal on *T*_1_WI and a high signal on *T*_2_WI.

### Lipoma

#### Clinical background

Lipoma, composed of adipose tissue, is a common etiology originating from subcutaneous tissue.^10^ It could be found anywhere in the human body such as internal organs, periungual skin, head and neck, and bladder.^11–13^ Although lipoma is a benign entity, some lesions show infiltrative features, mimicking malignancies.^11,14,15^ Lipoma is rarely found in the perineum.

#### Pathological findings

Macroscopic appearance shows that it is lobulated, well-circumscribed, and sometimes covered by a thin capsule. The cut surface is homogeneously yellow, with fibrous septa dividing into lobules. The tumor consists of a relatively uniform population of mature adipocytes arranged in sheets and/or separated into lobules by thin fibrous septa. Adipocytes and septal stromal cells lack cytological atypia. Moreover, lip blasts are not present (Supplementary Fig. 1).

#### MRI findings

On imaging, diagnosis can be easily achieved by having adipose tissue signals on all scanning sequences. Lipoma usually displays as a well-demarcated mass with a homogeneous high signal on both conventional *T*_1_WI and *T*_2_WI sequences. The low signal of lesion on the FS *T*_1_WI sequence will aid in differentiating lipoma from ES for the latter also have a high signal on the FS sequence. To be noted, lipoma sometimes needs to be distinguished from liposarcoma on imaging. In our institution, we do not meet the case with liposarcoma occurring in the female lower genital tract. Liposarcoma, a highly malignant tumor, often develops in retroperitoneal space in a larger mass with a more irregular margin. Post-contrast images will depict heterogeneously obvious enhancement in the early phase like most solid malignant tumors.

### Angiofibroblastoma

#### Clinical background

Angiofibroblastoma (AFB) is a rare tumor. When it was first reported in 1999, it was considered a unique etiology differentiated from other mesenchymal tumors of the skin.^16^ AFB is a benign and well-circumscribed myofibroblastic neoplasm. It usually arises in the pelvic-perineal region, especially in the vulva.^17^ About 10–15% of cases are located in the vagina.

#### Pathological findings

**Figure 3.**
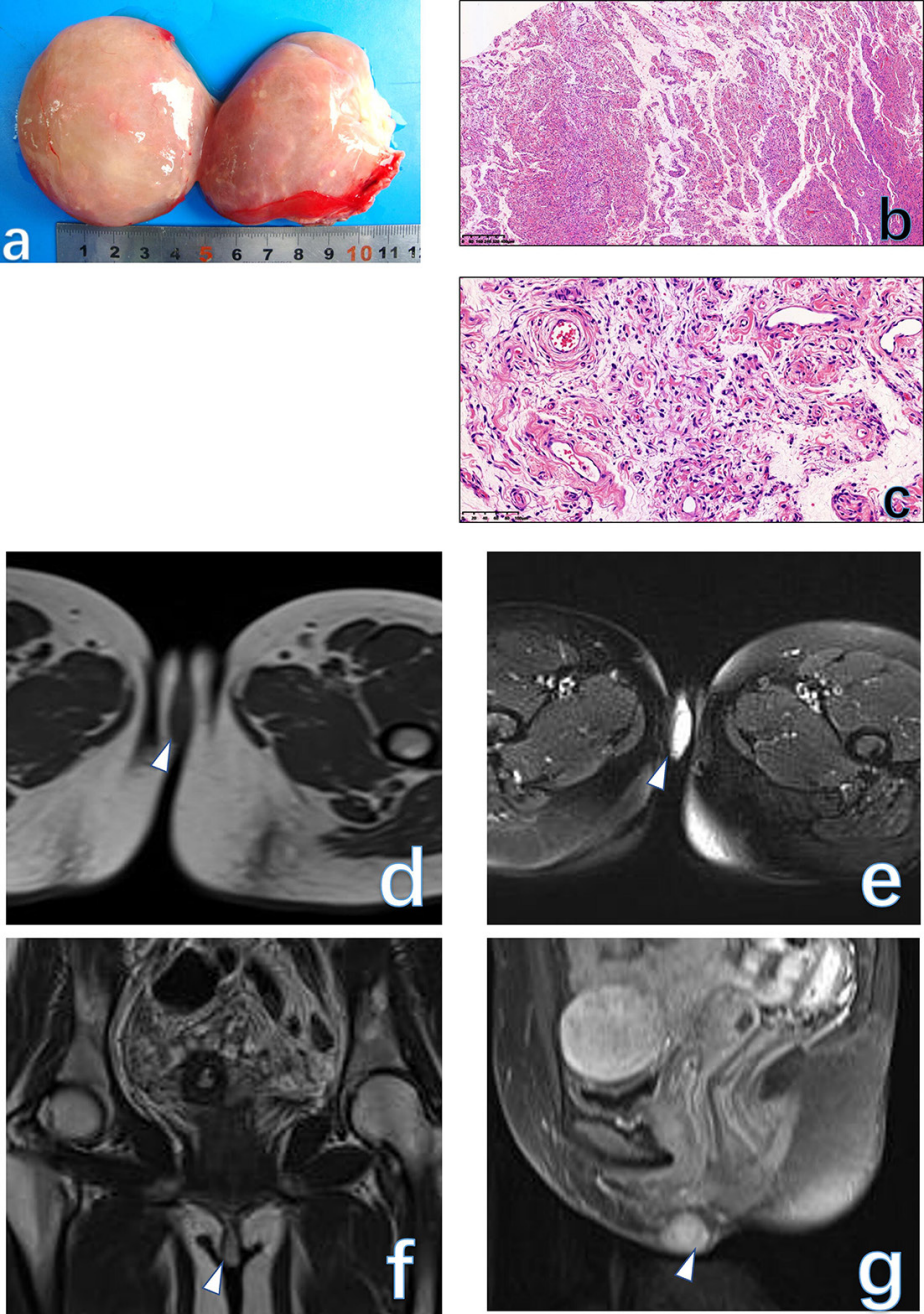
The gross specie shows that the tumor is well demarcated, and the cut surface is tan-white and solid (**a**). Low-power view of the tissue has alternating zones of cellularity and prominent small to medium-sized thin-walled vessels in an edematous matrix (**b**, HE × 40). Tumor cells are a plasmacytoid appearance, cluster adjacent to blood vessels in a loose edematous stroma, mitotic activity is absent. Some quantity of stromal collagen fibrils is present (**c**, HE × 200). The final histological diagnosis was AFB. A solid lesion (arrowhead) on the right side of the vulva was displayed as an isointense signal on *T*_1_WI (**d**) and a hyperintensity signal on *T*_2_WI (**e-f**). On post-contrast images (**g**), the lesion was enhanced vividly. AFB, angiofibroblastoma.

Some pathological features are listed below. First, the tumor is well demarcated with a tan-white and solid cut surface. Second, the diameter of AFB is usually less than 5 cm. Third, it has alternating zones of cellularity and prominent small to medium-sized thin-walled vessels in the edematous matrix, which are clustered adjacent to blood vessels in the loose edematous matrix. The morphology of tumor cells is spindled to ovoid, with a centrally to eccentrically located round nucleus, giving them a plasmacytoid appearance, mitotic activity is too rare to be identified. Some quantities of stromal collagen fibrils or sclerotic plaques are present (Figure 3A–C). Fourth, the tumor cells show strong and diffuse positive staining for desmin on immunohistochemistry, whereas there is only focal positivity for smooth muscle actin.^17,18^ Tumor cells consistently express estrogen receptors and progesterone receptors. Finally, the chance of recurrence is low.

#### MRI findings

There are no literatures reporting MRI findings of this rare etiology. In our case, the tumor appeared as a well-circumscribed solid lesion with an isointense signal on *T*_1_WI and hyperintensity signal on *T*_2_WI, while the tumor homogeneously enhanced similar to the normal uterus on post-contrast images (Figure 3D–G).

### Leiomyoma (LM)

#### Clinical background

Smooth muscle tumors are the most common soft tissue tumors of the vulva and vagina. Both vaginal and vulvar leiomyomas are rare entities in females of reproductive age [119]. The knowledge about these entities comes from case studies. Vulvar leiomyomas account for only 0.03% of all gynecologic neoplasms and 0.07% of all vulvar tumors.^19^ Vaginal LMs, identified as LM at a rare site, are usually found in the anterior vaginal wall in females between 35 and 50 years of age and are more common among Caucasian females.^20^ LM is the most common benign vaginal mesenchymal tumor and typically occurs in reproductive and pre-menopausal females.^21^ The tumors can arise anywhere in the vagina. Large tumors may cause pain, dyspareunia, bleeding, dystocia, and urinary tract symptoms.

#### Pathological findings

**Figure 4.**
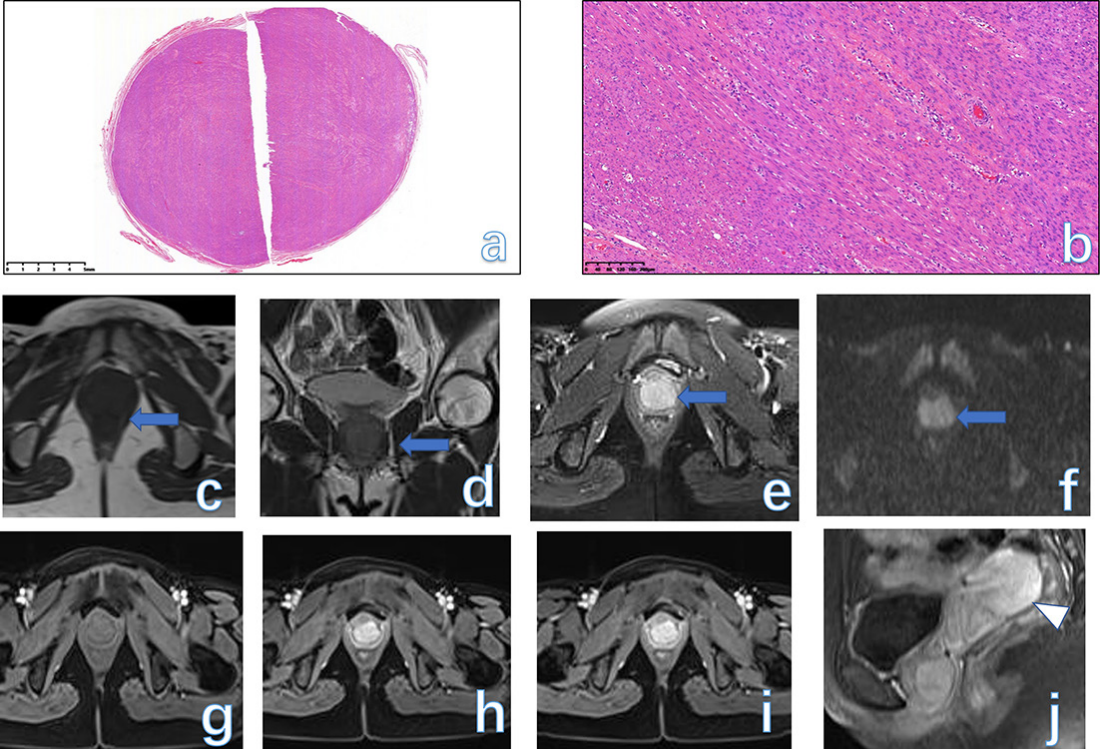
Low-power view shows a well-circumscribed tumor nodule composed of broad fascicles of the spindle cells (**a**, HE × 25). The spindle cells have bland cytological features, with elongated nuclei and fine nuclear chromatin (**b**, HE × 100). An oval, solid mass (arrow) occupied the lower segment of the vaginal wall with a homogeneous isointense signal on both *T*_1_WI (**c**),*T*_2_WI (**d**), and FS-*T*_2_WI (**e**). The mass appeared as a slightly high signal on DWI (**f**). On serial post-contrast images (**g-j**), the mass showed progressive enhancement similar to the normal uterus (arrowhead). The final pathology was LM in a 34-year-old female. DWI, diffusion-weighted imaging; FS, fat-saturated; LM, leiomyoma.

The gross and microscopic features are similar to their uterine counterparts. They are usually submucosal and composed of spindle cells or occasionally epithelioid cells.^22^ About 10% of tumors have myxoid foci, and few tumors have bizarre nuclei (Figure 4A–B, Supplementary Fig.2). The differential diagnosis of LM is usually with leiomyosarcoma (LMS).

#### MRI findings

**Figure 5.**
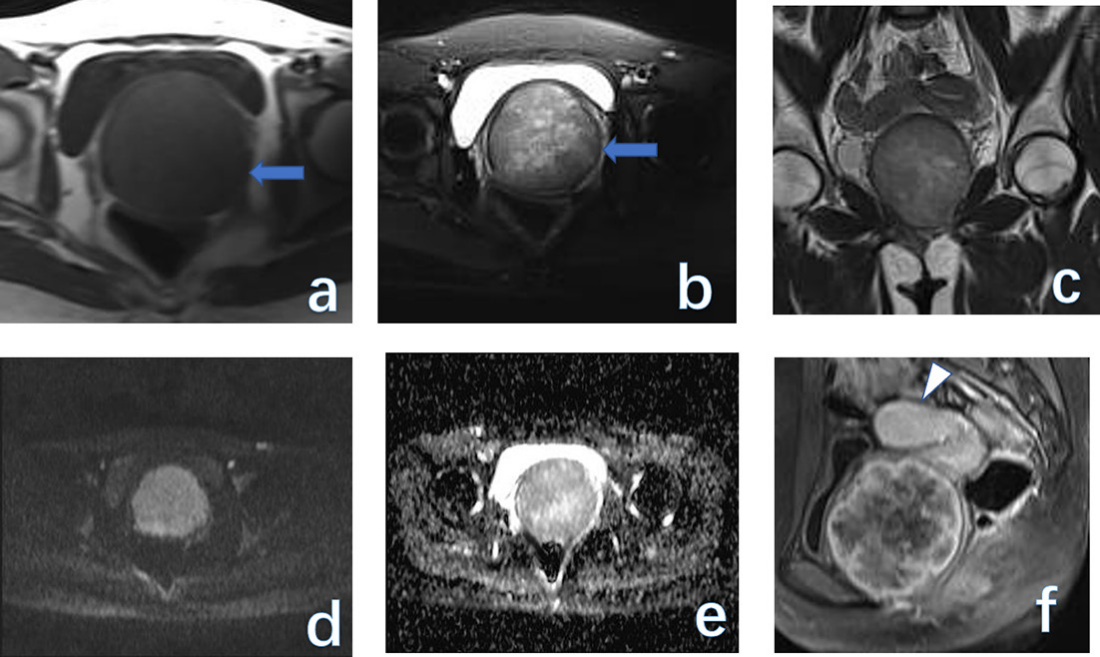
**(a)** 21-year-old young female with LM. A giant, solid mass (arrow) located at the anterior wall of the vagina. The mass had an isointense signal on *T*_1_WI (**a**) and a heterogeneous signal on *T*_2_WI (**b-c**). The mass showed a slightly restricted signal on both DWI (**d**) and ADC map (**e**). On the post-contrast image (**f**), the mass displayed marginal enhancement compared to the normal uterus (arrowhead). ADC, apparent diffusion coefficient; DWI, diffusion-weighted imaging; LM, leiomyoma.

On MRI, the key characteristic of vulvar or vaginal leiomyoma is the signal intensity similar to that of smooth muscle on *T*_2_WI.^23^ LM usually displays as a well-circumscribed mass with a homogeneously intermediate signal on both *T*_1_WI and *T*_2_WI. If some degeneration within the tumor occurs, then the foci of high signal on *T*_2_WI are also seen. On DWI, the tumor can show a mildly restricted signal. On contrast-enhanced MRI, the mass often shows obvious enhancement, which can be mistaken for malignancies^2^ (Figure 4C–J, Figure 5).

### Other benign conditions

Other entities might include Bartholin’s cyst, periurethral cysts, abscess, hematoma, lichen sclerosis, nerve sheath tumors, and so on for the vulva and perineum alone. Most of these lesions are accidentally found during complaints of other severe complications at the clinic. Most of the patients with benign etiologies will undergo conservative therapy and we cannot compare some connections. Among them, Bartholin’s cyst is the most common benign cyst in the major labium with a pure high signal on *T*_2_WI. A perineal hematoma is usually related to an invasive procedure with a mostly high signal on both *T*_1_WI and *T*_2_WI (Supplementary Fig.3).

### Malignant etiologies

#### Squamous cell carcinoma

##### Clinical background

Vulvar cancer is an uncommon gynecologic malignancy primarily affecting post-menopausal females. Squamous cell carcinoma (SCC) is the most common subtype among patients with vulvar cancer.^24^ Symptoms may include perineal pruritus, pain, a lump, or an ulcer. SCCs of the vulva account for 90% of vulvar cancers and 5% of gynecologic cancers.

##### Pathological findings

**Figure 6.**
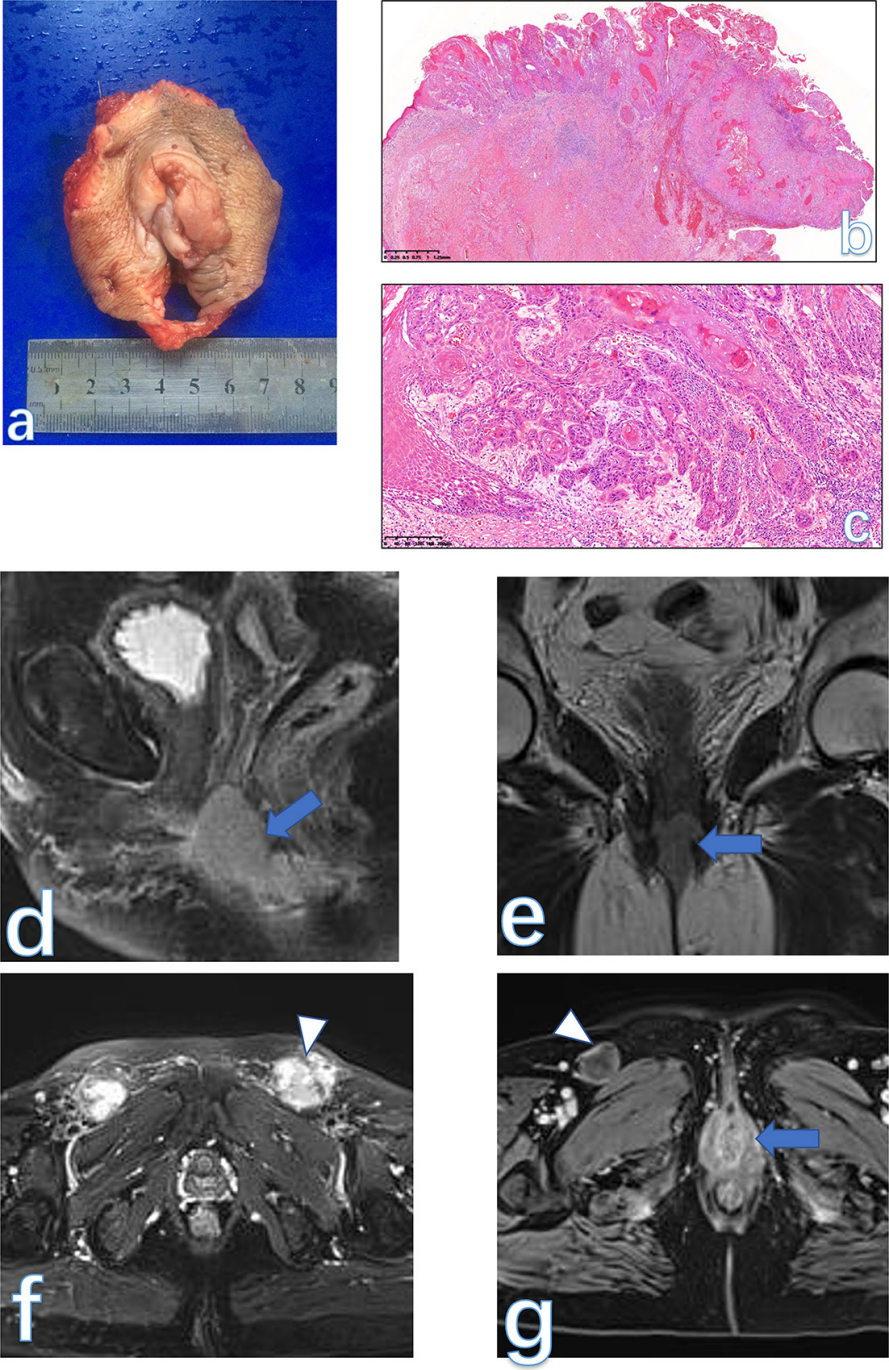
SCC forms an exophytic mass in the left labium majus and minus (**a**) that is associated with lichen sclerosus (white areas, right). Low-power view of SCC displays an infiltrative growth pattern (B, HE × 25). The tumor cells are well-differentiated keratinizing carcinoma with prominent pearl formation and numerous small nests of invasive tumor cells lie within a loose reactive stroma at the invasive border (C, HE × 100). In the center of the vulva, a mass with a homogeneously intermediate signal on *T*_2_WI (**d**) and *T*_1_WI (**e**) was seen on both *T*_2_WI and *T*_1_WI. Bilaterally inguinal nodes were detected on axial *T*_2_WI (**f**) and contrast-enhanced *T*_1_WI (**g**). Note, the mass enhanced and the necrotic components of the node (arrowhead) did not enhance. The final pathology was the SCC of the vulva (20% ki67 expression, FIGO IVB). SCC, squamous cell carcinoma.

SCCs include two subtypes: HPV-associated and HPV-independent. An HPV-associated pathway in approximately 50% of SCCs, usually in pre-menopausal females, leads to warty and basaloid SCC. It is commonly associated with HPV infection.^25,26^ The tumor cells typically show strong diffuse p16 staining (due to HR-HPV integration), but usually, wild-type p53 expression. A p53-associated pathway in approximately 40% of cases, which usually occurs among females in the seventh to eighth decades of life, leads to keratinizing SCCs. It is usually not related to HPV but associated with one or more of the causes such as differentiated vulvar intraepithelial neoplasia, lichen sclerosis, squamous cell hyperplasia, and p53 mutations (Figure 6A–C). Most tumors are p53 positive expression and exhibit TP53 mutations. However, p16 staining is usually absent or weak and focally present among those cases. HPV-independent vulvar SCC has a worse prognosis than HPV-associated vulvar SCC. Recurrence rates of HPV-independent vulvar SCC are higher than that of HPV-associated SCC, ranging from 12 to 37%.^4,27^

##### MRI findings

**Figure 7.**
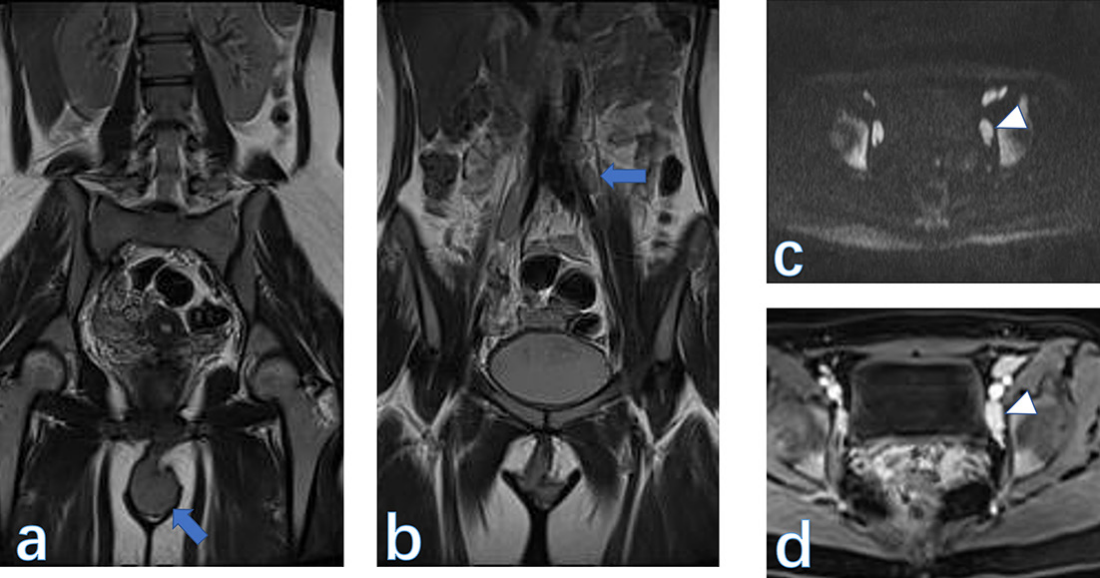
Coronal *T*_2_WI revealed the vulvar mass (A) and the enlarged nodes (B) beside the left iliac artery. The pelvic nodes (arrowhead) were depicted on DWI (C) and enhanced (D) obviously after contrast injection. The final pathology was squamous carcinoma of the vulva (70% ki67 expression, FIGO IIIC). DWI, diffusion-weighted imaging.

The tumors often appear as the focal thickness of the skin. The large tumor usually appears as soft tissue mass signals with homogeneous or heterogeneous enhancement after contrast agent injection (Figure 6D–G). Vulvar SCC almost always involves the skin surface and may be ulcerative. The mass has an intermediate signal on *T*_2_WI and a restricted diffusion signal on DWI. The primary purpose of imaging is to accurately identify and stage the disease. Staging and lymph node involvement are the most important prognostic factors. MRI has advantages in imaging enlarged nodes in both the pelvis and abdomen (Figure 7).^2,3,28^ In this respect, MRI has its own advantages in the preliminary stage including primary lesion contour, margin, and extent, in addition to both pelvic and abdominal nodes status. Axial MRI scans will help to delineate both parailiac and inguinal region nodes; while coronal MRI scans will facilitate the delineation of paraaortic nodes. MRI is the suitable modality to image tumors that are not confined to the vulva and perineum (usually with more than 2 cm in diameter and more than 1 mm stromal invasion). The boundary between the tumor and adjacent structures (the urethra and upper two-thirds of the vaginal) will help to precisely stage pre-operatively. In general, the suspected nodes in the pelvis with more than 10 mm in the short diameter now are considered as the cut-off value for potential malignant nodes. According to the newest FIGO stage, if any inguinofemoral node with more than 5 mm in diameter, then the tumor will be divided into FIGO Stage III or more.^3^

### Paget’s disease (PD)

#### Clinical background

Extramammary PD is a rare etiology that usually develops in apocrine gland-rich areas, such as the vulva, scrotum, and penis.^29^ It may present as a focal/multifocal lesion, which is difficult to be differentiated from erythema and eczema clinically.^30^ PD, accounting for approximately 1% of vulvar cancers, occurs in the late reproductive and post-menopausal age groups, with a median and mean age in the seventh decade. Intraepidermal recurrence occurs in up to 60% of cases due to incomplete excision. In most studies, the risk of recurrence does not correlate with resection margin status. Rarely, there is a progression to invasive PD during the follow-up period.

#### Pathological findings

**Figure 8.**
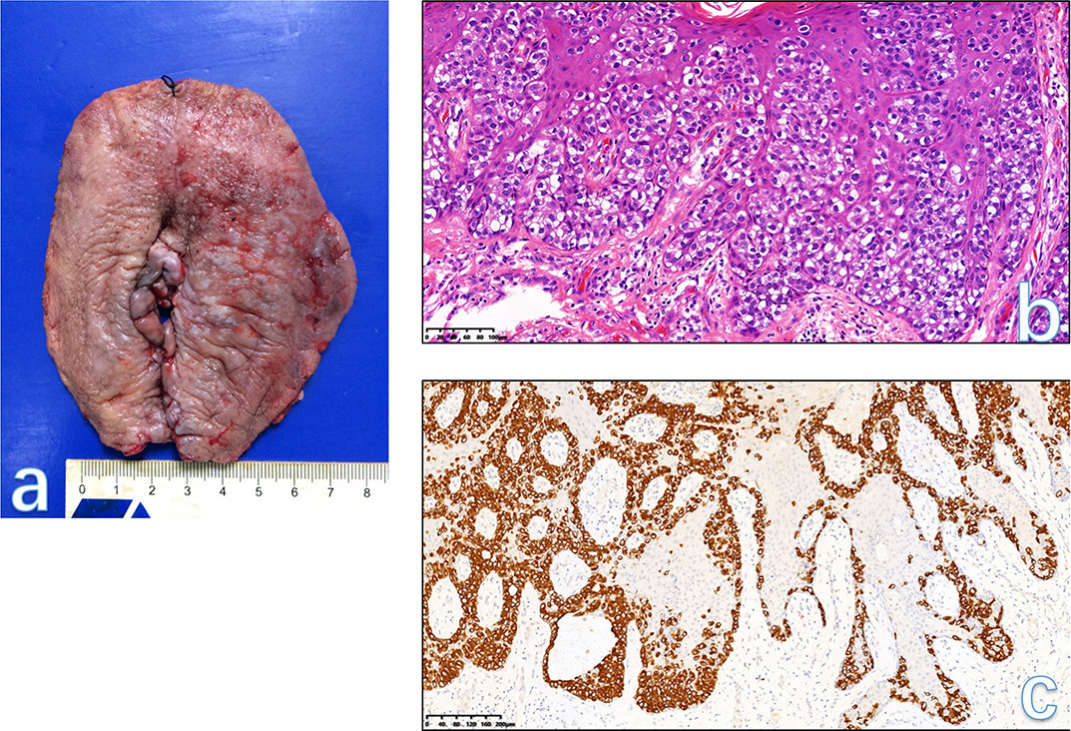
Vulvar PD extensively arises from both the left labium majus and labium minus with an ill-defined margin (A). Large round Paget cells with abundant pale cytoplasm and a large nucleus, frequently with a prominent nucleolus are present within the epidermis. Nests cells are predominantly basal distribution and extend into the more superficial layers of the epidermis (B). The atypical cells in PD are strongly positive for CK7(C). The focal thickness of vulvar tissue was seen on sagittal fat-suppressed *T*_2_WI (D, arrowhead) and enhanced mildly (E). The final histological diagnosis was vulvar PD. PD, Paget’s disease.

Vulvar PD is an *in-situ* adenocarcinoma of the vulvar skin, with or without underlying invasion. Secondary involvement of the vulvar skin by carcinoma of rectal, bladder, or cervical origin is designated “secondary PD”. Vulvar PD is thought to originate from pluripotent stem cells in the epidermis or skin appendages, arising from either the labium majus or labium minus and can extend to the extravulvar skin and/or involve the vaginal or cervical mucosa.^31^ PD typically presents as mild eminence and redness of the skin with ill-defined margins, compared with the adjacent normal skin. Paget cells are diffusely and strongly positive for CK7 (Figure 8), which is a good marker of *in situ* and invasive Paget cells. CK7 can distinguish Paget cells from hyperplastic and malignant squamous cells.^32^

#### MRI findings

On MRI, the lesions do not show any specific signs, usually only display as the focally thickening skin. In our hospital, the purpose of MRI for most cases of vulvar PD is to identify the pelvic condition and the potential enlarged nodes in both the abdomen and pelvis. Imaging diagnosis should depend mainly on both clinical signs and histological diagnosis.^5^

### Gestational trophoblastic neoplasia (GTN) disease

#### Clinical background

Gestational trophoblastic neoplasia (GTN) refers to gestational trophoblastic lesions that have the potential for local invasion and metastasis. The reported incidence varies between 1/600 in therapeutic abortions and 1/1000–1200 in normal pregnancies. Ultrasound is the first-choice modality in diagnosing GTN. Combined with an elevated hCG level for normal gestational age is highly suggestive of molar pregnancy or GTN.^33^

#### MRI findings

MRI is not routinely used in clinics to image GTN, mostly dedicated for GTN staging and evaluation of complex GTN. The common metastatic sites are the lungs (80%), vagina (30%), pelvis (20%), liver (10%), and brain (10%).^34^ The molar tissues may show a “grape appearance” in both the cavity and myometrium. If solid components consistently persist, then the pre-operative diagnosis should be cautiously diagnosed and needs to be differentiated from an incomplete miscarriage or an ectopic pregnancy as vivid enhancement may be seen in all these abnormal etiologies. For post-treatment evaluation of GTN on MRI, the multicystic lesions with hemorrhagic components displaying as a high signal may present as the primary manifestation. The enlarged vessels surrounding the lesion may appear as flow void signals in every protocol of MRI. The hemorrhagic components often show a high signal on *T*_1_WI. MR angiography or venography helps image potential coexistent vascular malformations (arteriovenous malformation or fistula). These lesions together appear as the blood pool on maximum intensity projection reconstructed images (Supplementary Fig. 4).

### Leiomyosarcoma (LMS)

#### Clinical background and pathological findings

**Figure 9.**
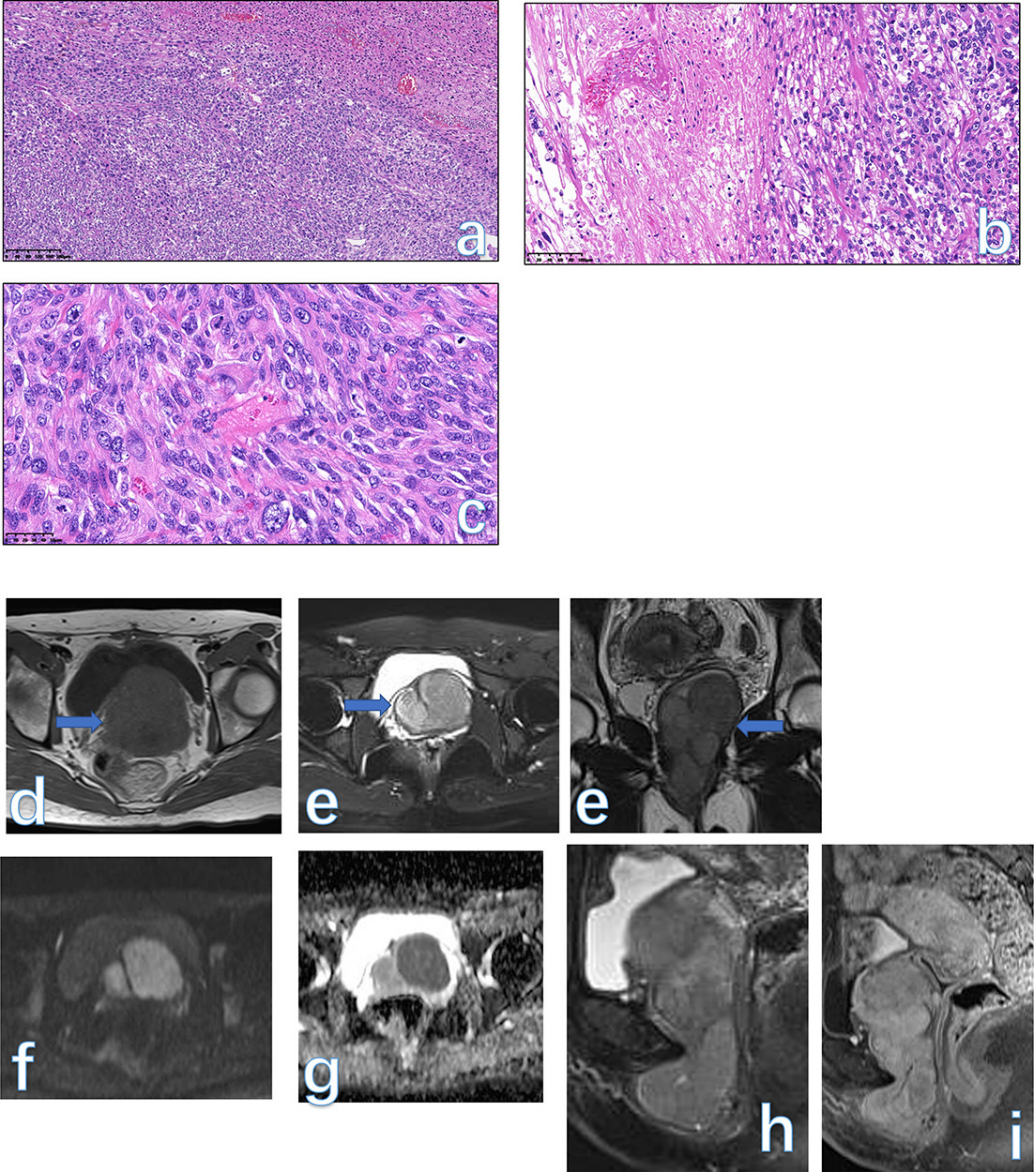
A spindle-cell type LS. The tumor is the cellular type with a fascicular growth and the enlarged hyperchromatic nuclei are visible at low-power magnification (A). Coagulative tumor cell necrosis (B) with a sharp interface between viable (right) and non-viable tumor (left) shows significant pleomorphism. Mitotic figures are easily identified (C). An oval, solid lesion (arrow) centered in the vagina was mainly seen with an intermediate signal on *T*_1_WI (D) and a high signal on *T*_2_WI with and without fat-saturated technique (E ). The mass showed a restricted signal on diffusion-weighted imaging (F) and a low signal on the apparent diffusion coefficient map (G). The mass grew extensively along the whole vagina and vulva (H) and showed moderate enhancement (I) after contrast injection. The final histological diagnosis was LS in a 36-year-old female. LS, leiomyosarcoma.

LMS is the most common sarcoma of the lower genital tract, arising more frequently in the vulva than in the vagina. Patients are usually identified in their fifth to sixth decade of life at diagnosis. LMS account for approximately 2% of all vaginal malignancies. LMS, a malignant neoplasm, is composed of cells showing smooth muscle differentiation.^35^ LMS includes three subtypes: spindled, epithelioid, and myxoid.^36,37^ There is a solitary mass, usually with a diameter of more than 5 cm, and circumscribed or infiltrative borders. The cut surface is soft or firm and fleshy white-tan-gray with variable hemorrhage, necrosis, and cystic change. Classically, the criteria for malignancy are at least two of the three features: moderate to severe cytological atypia, mitotic count >10 mitoses/10 HPF of 0.53 mm in diameter and 0.22 mm^2^ in area, and tumor cell necrosis^35,37,38^ (Figure 9A–C). Vulvovaginal LS are high-grade malignancy, with frequent local and distant recurrence. A significant percentage of patients ultimately die of the disease.^39,40^

#### MRI findings

Since LMS often develops after pelvis radiation therapy, complete clinical history would help to establish a proper diagnosis. Further, LMS usually occurs in the posterior wall of the vaginal and exhibits as an ill-marginated mass.^1^ MRI is not ideal for differentiating benign from malignant forms in doubtful cases, because on the one hand, some benign-appearing lesions may be aggressive over time, and on the other hand, the imaging signs could overlap to some extent between the two etiologies.^2,3,5^ On post-contrast images, both etiologies could be wholly or marginally enhanced, really posing a challenge to establish a correct diagnosis before surgery (Figure 9D–I). Relatively, necrosis and hemorrhage inside the LMS more often develop owing to the malignant nature. Further, at the later stage, the tumor also invades the adjacent tissues (such as bladder, urethra and rectum). At this time, MRI will provide valuable information on the pre-operative stage of the tumor.

### Rhabdomyosarcoma

#### Clinical background

Rhabdomyosarcomas (RMS) is the most common vulvar malignancy in pediatric patients (median age of onset is 16-years-old).^28^ RMS, occurring in the lower genital tract, is a rare entity that is mostly reported in case of report studies.^41,42^ It is reported that the 5-year disease-specific survival rate is approximately 62.5% among females with RMS. RMSs are a family of malignant mesenchymal tumors exhibiting skeletal muscle differentiation. They include three subtypes: embryonal, alveolar, and pleomorphic. Embryonal RMS is the most common vaginal malignancy in infants and young children. Approximately, 90% of the patients are those in the first 5 years of life period (mean 1.8 years); young adults and post-menopausal females may also be affected.^43^ The tumors present as vaginal bleeding and vaginal mass which is typically soft, edematous, nodular, papillary, or polypoid (sarcoma botryoides), often with introital protrusion.

#### Pathological findings

**Figure 10.**
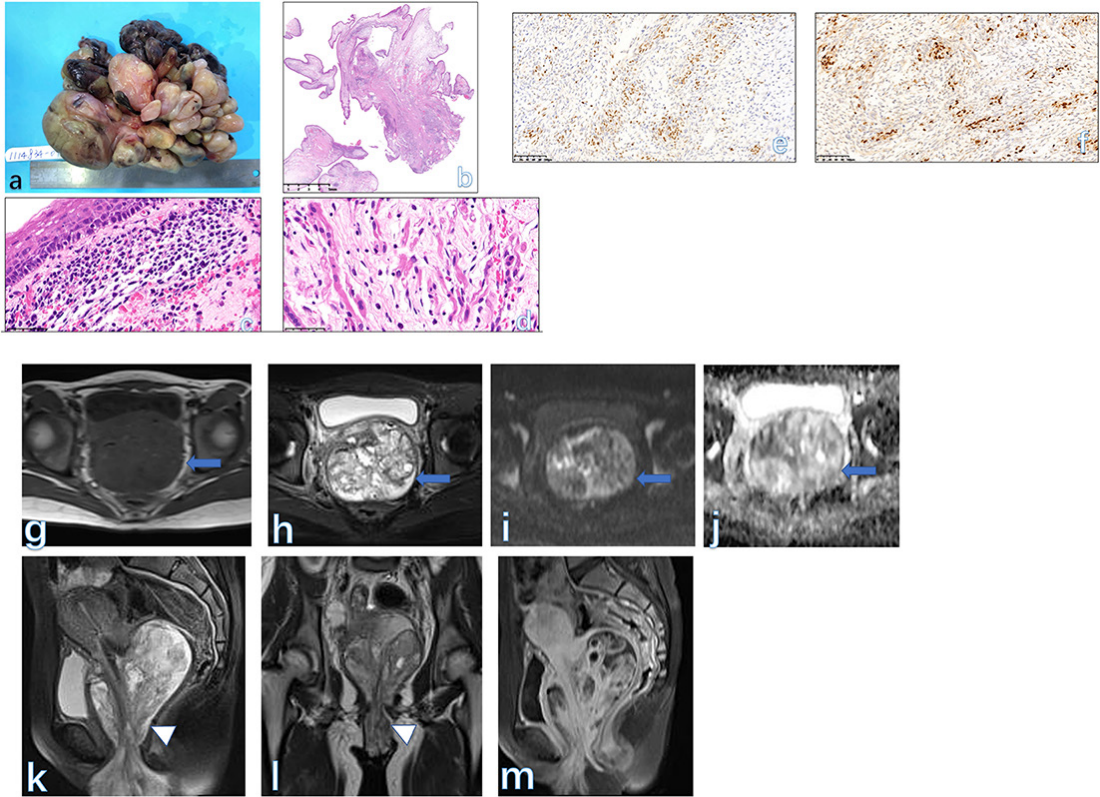
A vaginal mass is typically a soft, edematous, polypoid botryoid mass (A). Polypoid fronds of the botryoid form are typically seen on histological imaging (B). The cambium layer (subepithelial condensation of stroma) is present (C, HE × 200). The strap-shaped rhabdomyoblasts have eosinophilic cytoplasm with cross-striations (D, HE × 400) and positive nuclear staining for myoD1 (E) and myogenin (F). A giant mass (arrow) occupied the whole vaginal cavity and extended downwards to the vulva (arrowhead). The mass is mainly displayed as an isointense signal on both *T*_1_WI (G) and *T*_2_WI (H). The tiny small cysts were seen within the lesion. The tumor did show a restricted signal on DWI (**I**) and ADC map (J). The sagittal (K) and coronal (L) *T*_2_WI more clearly showed the contour of the mass. On post-contrast images (M), the tumor enhanced similarly to the normal uterus. The final pathology was RMS in a 15-year-old girl. ADC, apparent diffusion coefficient; DWI, diffusion-weighted imaging; RMS, rhabdomyosarcomas.

There is a densely cellular zone (cambium layer) of primitive, small, mitotic cells under the squamous epithelium that may be invaded by tumor cells. Beneath the cambium layer is a hypocellular edematous zone of similar small cells and rhabdomyoblasts; foci of hyaline cartilage may also be seen. The rhabdomyoblasts, which may be sparse, vary from round to strap-shaped. They have eosinophilic cytoplasm with cross-striations in most cases. Immunoreactivity of desmin and more specifically, skeletal muscle markers (myoglobin, myogenin, myoD1) facilitate the diagnosis (Figure 10A–F). The tumors can invade local tissues and metastasize to regional lymph nodes or distant sites.

#### MRI findings

**Figure 11.**
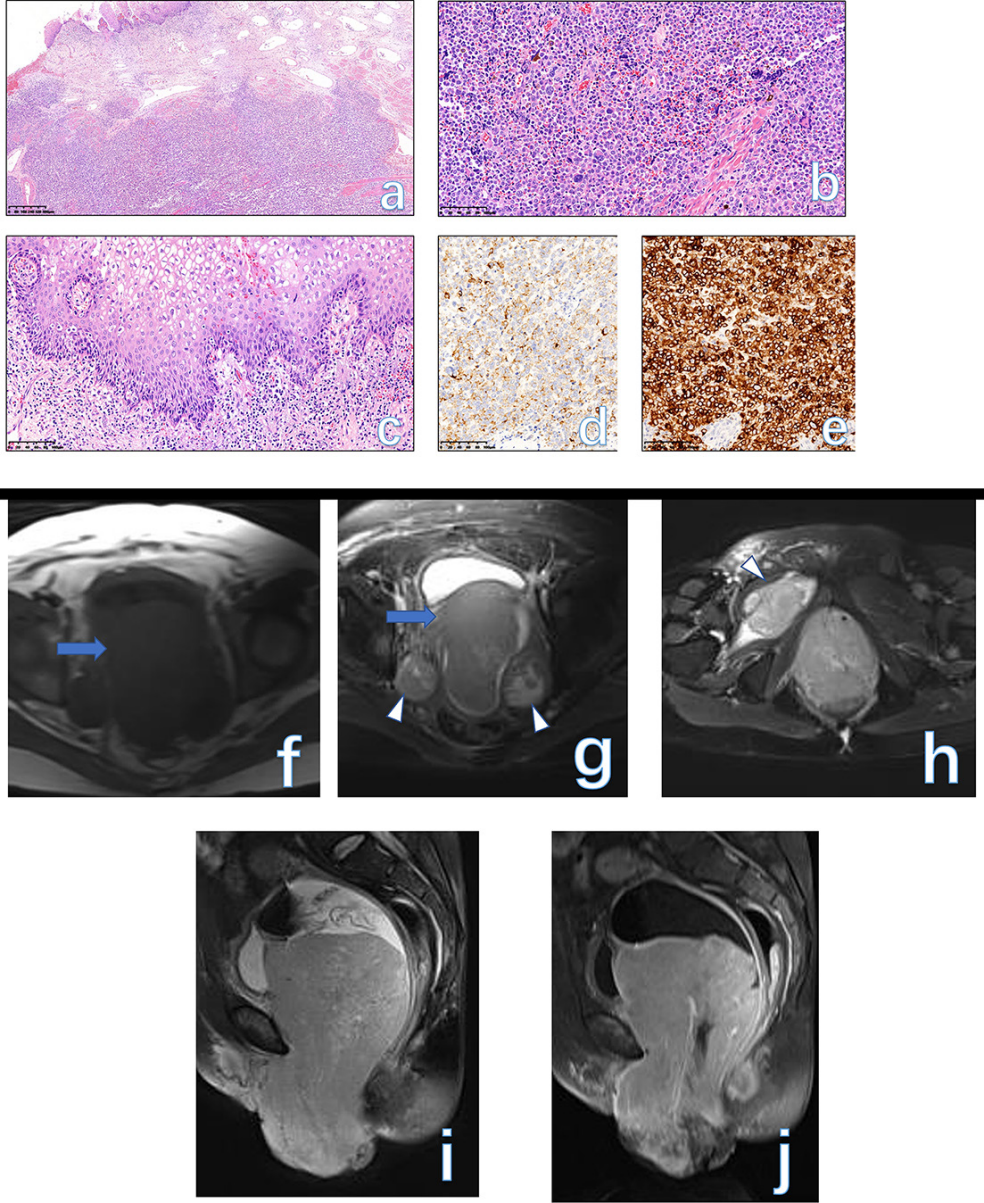
Low-power view shows sheets of large malignant melanocytic cells underneath the epidermis (A, HE × 40). Pleomorphic epithelioid melanocytic cells, irregular nuclear contour, conspicuous nucleoli, and mitotic figures are more commonly observed on the high-power view of the slide. Cytoplasmic melanin was focally present (B, HE × 400). Lentiginous *in situ* malignant melanoma with pagetoid spread component involves basal layers of the vaginal squamous mucosa (C, HE × 400). Melanocytic cells show positive stains for both HMB45 (D) and Melan-A staining (E, HE × 200). A giant mass (arrow) occupied the whole vaginal and vulvar area with a homogeneous isointense signal on both *T*_1_WI (F) and *T*_2_WI (G). The enlarged nodes (arrowhead) were seen on both the bilateral pelvic wall (G) and piriformis space (H). The mass showed moderate enhancement (**I–J**) after contrast injection. The final histological diagnosis was vulvar melanoma in a 54-year-old female.

Vaginal RMS often develops widely, filling the vagina and invading the adjacent anatomic structures (vulva and bladder). RMS could involve the cervix, vagina, and vulva. They generally appear as cystic-solid masses with isointense signals on both *T*_1_WI and *T*_2_WI, which is confirmed by histological findings. Heterogeneous enhancement is often observed on MRI of RMS (Figure 11G–M), in which hemorrhage or calcification are not common signs.^44^

### Melanoma

#### Clinical background

Vulvar melanoma (VM) accounts for 1% of all melanomas in females and 5% of all vulvar malignancies.^45–47^ Compared with cutaneous melanoma, the prognosis for VM is still worse (5-year overall survival rate: 92 *vs* 47%). Primary VM is also an extremely rare tumor of the lower genital tract, accounting for only 0.3–0.8% of all melanomas in females.^47^ Malignant melanoma is a malignant melanocytic neoplasm arising in a non-cutaneous site of the female genital tract, usually in the vulva or vagina (lower third of the vagina and anterior wall) and rarely in the cervix. Malignant melanoma accounts for 5% of vulvar and vaginal malignancies and is most common in older females with a mean onset age of 65 years.

#### Pathological findings

As many as 70% of patients have Stage III or IV disease at presentation. VMs usually present as pigmented plaques, but amelanotic ulcerated nodules are also common.^46^ They usually present as large, ulcerated, and nodular masses; Moreover, they often involve the cervix. The tumor is usually formed by sheets or expansive nodules of large pleomorphic epithelioid or (less commonly) spindle malignant melanocytic cells. Melanin production is variable but usually focally present within melanoma cells. Immunoreactivity of S100, SOX10, HMB45, and Melan-A (MART1) facilitates the diagnosis (Figure 11). The overall survival is relatively poor for patients with primary melanoma of the female low genital tract. Increased tumor thickness, ulceration, and a high mitotic count on histological imaging are poor prognostic factors for vulvovaginal melanoma.

#### MRI findings

On MRI, the signal intensity of vulvovaginal melanoma may be related to the amount of melanin. Melanoma can demonstrate an iso- to hyperintense signal on *T*_1_WI and a relatively low signal on *T*_2_WI as a result of the paramagnetic effect of melanin. It is reported that nearly 5% of all vulvovaginal melanomas are amelanotic. Amelanotic melanoma of the vagina may be mistaken for other primary vaginal malignancies (Figure 11F–I, Supplementary Fig.5). If vulvar lesions are small, they need to be differentiated from SCC owing to the overlapping imaging signs (Table 1). The final diagnosis relies on a biopsy or histological diagnosis.^48^ Vaginal melanoma needs to be differentiated from SCC which is often ulcerative and located in the upper third of the vagina and posterior wall. VM often occurs in the clitoris, while SCC develops in the labia majora. If tumor bleeding occurs in the tumor, then a high signal within the lesion can be detected. At this time, perineal endometriosis should be included as the differential diagnosis for the latter also have a high signal on both *T*_1_WI and *T*_2_WI. For melanoma staging, it can be regarded as vulvovaginal cancer FIGO stage. There was a report that vaginal melanoma with less than 3 cm in diameter has a better prognosis.

**Table 1.** Summaries of MR major imaging features in the reported literatures for uncommon vulvar and vaginal malignancies

Study	Numbers and pathology	Component	Signal	Enhancement
LM and LS				
Egbe, et al.^23^	1,LM	Solid	Intermediate signal on both *T*_1_WI and *T*_2_WI	Homogeneous
Shah M, et al.^21^	1,LM	Solid	NA	NA
Ours	3,LM	Solid	Intermediate signal on both *T*_1_WI and *T*_2_WI	Two with homogeneous and one with inhomogeneous
Aljehani AM, et al.^36^	1, LS	Solid	Heterogeneous signal intensity on *T*_2_WI	NA
Akrivi, et al.^35^	1, LS	Solid	NA	NA
Ours	1, LS	Solid	Intermediate signal on both *T*_1_WI and *T*_2_WI	Homogeneous
RMS				
Sun F, et al.^44^	2, RMS	Cystic-solid	Isointensity on *T*_1_WI and hyperintensity signal on *T*_2_WI	Inhomogeneous
Solomon LA, et al.^43^	1, RMS	Cystic	Iso-hyperintensity signal on *T*_2_WI	NA
Ours	1, RMS	Cystic-solid	Isointensity on *T*_1_WI and iso-hyperintensity signal on *T*_2_WI	Inhomogeneous
Melanoma				
Stein R, et al.^3^	1,melanoma	Solid	Mild hyperintensity on *T*_1_WI and hyperintensity signal on *T*_2_WI	Homogeneous
Ours	2, melanoma	Solid	Iso-hyperintensity on *T*_1_WI and mild hyperintensity signal on *T*_2_WI	Homogeneous

LM, leiomyoma; LS, leiomyosarcoma; NA, non available.

In summary, vulvar and vaginal lesions cover a wide spectrum of etiologies with mimicking imaging findings. Owing to the high soft tissue resolution, MRI helps delineate the extent of the primary lesion (especially for malignancies), determine the etiology, and evaluate the metastatic lesions. Superficial lesions cannot be satisfactorily displayed on MRI and the role of MRI is to determine the extent of lesions and coexistent lesions. Endometrial cysts in the lower genital tract and peritoneal area should be included as the differential diagnosis other than the most common lesions (such as Bartholin gland cyst and vaginal cyst). For solid masses in these areas, LM is the common benign etiology with a clear margin; while SCC is the most common malignant tumour, in which MRI has a critical role in staging. LMS and VM are also needed to be considered when having a large mass with an irregular margin (LMS) and high signal on plain *T*_1_WI (VM). To be noted, even if no high signals on *T*_1_WI, VM is still not definitely excluded. Post-contrast images may provide limited information in differentiating between LM and LMS because both trilogies enhance moderately or vividly. RMS is the most common vulvar malignancy in pediatric patients with the characteristic feature: a polypoid botryoid mass on post-contrast MRI. Clinical signs and symptoms are still necessary to establish a proper diagnosis. The final diagnosis is based on histological findings. To reasonably utilize an MRI scan pre-operatively will aid in decision-making and individual treatment plan.

## Key points

- Knowledge about vulvar and vaginal pathology will help establish a proper pre-operative MRI diagnosis.
- MRI plays an important role in staging malignant tumors located in the lower genital tract.
- The diagnosis of small and tiny lesions developing in the perineum relies on histological findings thereafter.
